# Chronic stress modulates the expression level of leptin and leptin receptors in the hypothalamus of male rats with a history of maternal stress

**DOI:** 10.1016/j.bbih.2024.100895

**Published:** 2024-10-28

**Authors:** Roya Hosseini, Sara Emadian, Manijeh Dogani, Touba Ghazanfari, Nayere Askari

**Affiliations:** a Department of Biology, Faculty of Sciences, Shahid Bahonar University of Kerman, Kerman, Islamic Republic of Iran; b Immunoregulation Research Center, Shahed University, Tehran, Islamic Republic of Iran

**Keywords:** Chronic stress, Maternal stress, Social instability stress, Unpredictable stress, Leptin, Anxiety

## Abstract

The activity of different neurotransmitter pathways in the hypothalamus controls the stress response. Meanwhile, leptin is known as an effective mediator in the stress response, and its serum and brain levels change when exposed to stressful factors. In this study, the effect of chronic social instability stress (INS) and chronic unpredictable stress (CUS) on anxiety-like behavioral responses and the level of expression of leptin and its receptor in the brain of male Wistar rats that were under maternal stress (MS) were investigated. Grouping: control (n = 7), MS (n = 7), INS (n = 7), CUS (n = 7), MS + INS (n = 7), MS + CUS (n = 7). Forced swimming, elevated plus-maze, and open field tests were used to check anxiety-like behaviors. Next, the mRNA expression of leptin and its receptor in the hypothalamus was measured by Real-Time PCR. According to the results, adult rats with maternal stress showed an increase in their anxiety-like behaviors faced with the stress of chronic social instability and chronic unpredictable stress (compared to the groups that only received adult stresses). Also, the hypothalamic expression of leptin decreased, but we saw an increase in the expression of hypothalamic leptin receptors in INS, CUS, and MS groups and a decrease in MS + INS and MS + CUS groups. Results of this research suggest that leptin plays a role as an effective mediator in the occurrence of central and behavioral changes caused by maternal stress. In other words, it can be effective in changing resilience in the face of adult stress.

## Highlights

• Adult male rats with a history of maternal stress are more sensitive and less resilient in response to adult stresses.
• Hypothalamic leptin expression decreases in adult male rats with a history of maternal stress.
• Leptin plays a role as an effective mediator in the occurrence of central and behavioral changes caused by maternal stress.

## 1 Introduction

Stress impacts both the physical and mental health of humans. Regardless of age, gender, ethnicity, or religion, no one is immune to stress. Alarming statistics about stress highlight its widespread prevalence. According to a report from the American Institute of Stress, in 2022, approximately 33% of Americans experience severe stress, 77% experience stress that directly affects their physical health, 73% experience stress that impacts their mental health, and 48% experience stress that disrupts their sleep ('www.stress.org' 2024).

The body's response to stress is specific and varies based on factors such as the duration of stress (acute or chronic) (Chu et al., 2024), the type of stress (social, food, etc.) (Del et al., 2018), the stage of life (fetal, neonatal, puberty, or old age) (Mousikou et al., 2023), gender, and species of the animal (Rincón-Cortés et al., 2019; Russell and Lightman, 2019). Studies have shown that if a mother experiences depression or anxiety during pregnancy, she is at an increased risk for various adverse physical and psychological outcomes in her child. These known outcomes may include behavioral problems (van Bodegom et al., 2017), symptoms of attention deficit hyperactivity disorder (ADHD) (Vizzini et al., 2019), and impaired cognitive development (Martucci et al., 2021). These disorders can reduce a person's resilience to adult stress and lead to disability in various aspects of their personal and social life (Davis and Narayan, 2020).

Many biomarkers have been proposed for stress, with the majority, such as cortisol depending on the hypothalamic-pituitary-adrenal axis (HPA) (García-León et al., 2019). Leptin, on the other hand, is an independent stress biomarker (as it is not synthesized in the HPA axis) and decreases after acute stress (Bouillon-Minois et al., 2021). This peptide is mainly produced in white adipose tissue and to some extent in the brain, and it has a noticeable effect on the central nervous system. According to the literature, the expression level of leptin receptors is regulated under the influence of chronic stress (Xiao et al., 2020). The receptors are primarily expressed in the paraventricular nucleus (PVN), arcuate nucleus (ARC), ventral tegmental area (VTA), ventral and dorsolateral regions of the hypothalamus, anterior pituitary, adrenal cortex, and in other brain areas such as the hippocampus, and frontal cortex (Seoane-Collazo et al., 2020). Leptin receptors are mainly located in the ARC nucleus, which produces pro-opiomelanocortin (POMC) (Dodd et al., 2015). POMC is the direct precursor of three major pathways: the melanocyte-stimulating hormone axis, which regulates appetite (Wu et al., 2023); the adrenocorticotropic hormone (ACTH) axis, which regulates the secretion of glucocorticoids and is primarily involved in the stress response (Harno et al., 2018); and the β-endorphin axis, which produces endogenous opioid peptides (Nakamoto and Tokuyama, 2023). Additionally, leptin plays a role in regulating emotional behaviors. Studies have shown that systemic administration of leptin produces anti-anxiety effects, and the removal of leptin receptors in midbrain dopaminergic neurons increases anxiety-like behaviors (de Vrind et al., 2021; Liu et al., 2016).

In this study, we examine the effect of maternal stress and adult stress on the severity of anxiety behaviors. We investigated the stress markers, leptin, and cortisol, about maternal stress and 2 types of adult stress (social instability stress and unpredictable stress). According to the type of induced stress, the expression changes of these markers and their receptors in the hypothalamus were measured as a response to a specific type of stress. In addition, we investigated and compared the anxiety-like behaviors caused by the induction of various types of stress and the subsequent change in leptin responsiveness.

Based on the contents mentioned, this study aims to investigate the impact of chronic stress (maternal and adult stress, including social instability stress and unpredictable stress) on the levels of cortisol and leptin, which are markers of stress. Additionally, we will examine whether there are alterations in the expression of leptin receptors in the hypothalamus (a key regulator of anxiety responses) and assess anxiety-like behaviors in response to stress. Changes in the levels of these markers and their receptors' expression in the hypothalamus will be measured in response to a specific type of stress. Furthermore, we will compare anxiety-like behaviors induced by different types of stress and the resulting changes in leptin responsiveness.

## 2 Materials and methods

### 2.1 Animals

Ten male and twenty female adult Wistar rats (170–185 g) were purchased from the Neuroscience Research Center of Kerman. Before the experiments, the rats were acclimated to their new environment for two weeks (12:12 light/dark cycles - free access to water and food - 20–25 °C - 45 ± 5% humidity). Following animal care guidelines, the rats were purchased from the closest possible center to minimize transportation distance to the animal house. Transferring was done with standard conditions. Once settled, the animals were housed together, with daily checks during the adaptation period to ensure their well-being. Any rats that displayed aggression towards each other were separated to prevent harm. Also, The cages were cleaned three times a week.

*Ethical statement*: this study was conducted following the rules of the National Institutes of Health and Care Guidance and the ethics committee of Shahed University (IR.SHAHED.REC.1397.046).

### 2.2 Experimental Design and grouping

**Fig. 1:**
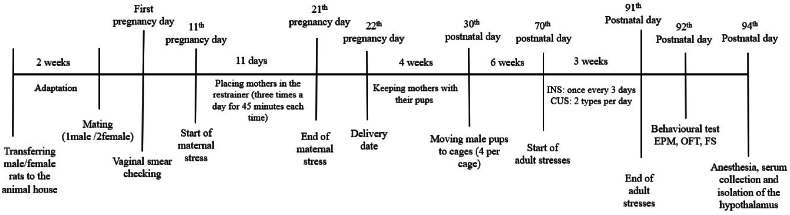
**Experimental Design.** This figure illustrates the timeline of the experiment, starting from the rat's acclimatization to the decapitation date and hypothalamus isolation (spanning approximately 18 weeks). INS refers to chronic social instability stress, and CUS to chronic unpredictable stress. EPM stands for elevated plus-maze test, OFT for open field test and FS for forced swim test.

The details of the experiment design are shown in Fig. 1 (Fig. 1).

Grouping: Based on previous similar studies, we considered a minimum of 7 rats (n = 7) for each study group (Abdelhamid et al., 2020; Kucukler et al., 2021). Male rats at the age of 3 days postnatal or 4 weeks old were randomly assigned to one of the following 6 groups:

1) control (n = 7)
2) maternal stress (n = 7)
3) chronic social instability stress (n = 7)
4) chronic unpredictable stress (n = 7)
5) maternal stress + chronic social instability stress (n = 7)
6) maternal stress + chronic unpredictable stress (n = 7)

### 2.3 Maternal stress/prenatal stress

During the mating phase, one male rat and two female rats were placed in each cage at 8 p.m. Twelve hours later, at 8 a.m., the formation of vaginal plaque was checked. To ensure accuracy, a smear was taken and examined under a microscope to confirm the presence of sperm. Once the first day of pregnancy was determined, mothers were placed in a restrainer (which restricts movement but does not affect breathing) from the 11th day of pregnancy until delivery on day 22. They were restrained three times a day at 9 a.m. (±2 h), 12 p.m. (±2 h), and 5 p.m. (±2 h), each time for 45 min. The variation in timing was used to prevent habituation.

### 2.4 Chronic social instability stress (INS)

Adult male rats (10 weeks old) underwent 3 weeks (21 days) of daily social instability stress. Every three days at 9 a.m. (±2 h), the rats were placed in a new cage with different roommates, involving a switch of two out of five rats.

### 2.5 Chronic unpredictable stress (CUS)

Adult male rats (10 weeks old) were subjected to various types of stressors (two types daily) for 21 days, beginning at 9 a.m. (±2 h). The stressors were administered following the schedule outlined in the table below (Table 1):

**Table 1:** 21 Days protocol for chronic unpredictable stress induction.

**Day**	**1**	**2**	**3**	**4**	**5**	**6**	**7**	**8**

**Stressor**	15 min forced swim (20 °C), 1 min tail pinch	12 h cage tilting (45 °C), 1 h cage rotation	reversal of the light/dark cycle, 1 h cold room (4 °C)	12 h wet bedding, crowded cage	24 h food deprivation, 1 h restraint	12 h cage tilting (45 °C), crowded cage	24 h water deprivation, 1 h cold room isolation	reversal of the light/dark cycle, 1 min tail pinch

**Day**	**9**	**10**	**11**	**12**	**13**	**14**	**15**	**16**

**Stressor**	cold room (4 °C), 1h cage rotation	24 h water and food deprivation, 12 h cage tilting(45 °C)	15 min forced swim (20 °C), 1 h restraint	reversal of the light/dark cycle, 24 h food deprivation	1 min tail pinch, cold room (4 °C)	24 h water deprivation, 1 h restraint	12 h wet bedding, 12 h cage tilting (45 °C)	1 h cage rotation, reversal of the light/dark cycle

**Day**	**17**	**18**	**19**	**20**	**21**			

**Stressor**	1 h restraint, crowded cage	12 h wet bedding, 1 min tail pinch	reversal of the light/dark cycle, 12 h cage tilting (45 °C)	15 min forced swim (20 °C), 24 h water deprivation	1 h cage rotation, crowded cage			

### 2.6 Behavioral assays

#### 2.6.1 Elevated plus-maze test (EPM*)*

The first behavioral test – was conducted the morning after the stress periods ended.

This behavioral model is one of the most well-known methods for measuring anxiety-related behavior. The device consists of a plus-shaped wooden maze with 2 open arms and 2 closed arms, each with dimensions of 50 cm. At the intersection of the four arms, there is a 10 × 10 cm square. The maze is elevated 50 cm from the ground by four bases, with a lamp (40–55 lux) positioned on the top to light up the arms. The animal was placed in the center square of the maze, facing the open arm, and allowed to move freely for 5 min. The number of entries and time spent in the open arms are recorded by the ANY-maze software (2020 version).

#### 2.6.2 Open field test (OFT)

The second test – was conducted 1 h after the EPM.

This test is a widely used method in behavioral research to assess motor activity levels, exploratory behaviors, and anxiety-related behaviors in a new environment. A black square box (70 × 70 × 30 cm) divided into 9 imaginary squares is used for this test. The animal was allowed to move freely in the box for 5 min, and the number of entries and time spent in the central zone (40 × 40 cm) were recorded by a camera.

#### 2.6.3 Forced swim test (FS)

The third test – was conducted 1 h after the OFT.

This test is used to measure anxiety and depression levels in animals such as rats. The animal was placed in a glass cylinder with a height of 40 cm and a diameter of 25 cm, filled with water at 25 °C. The animal's movements were recorded by the camera for 5 min and then evaluated by software. The duration of vertical and horizontal movements as well as the duration of immobility were measured.

### 2.7 Molecular analysis

#### 2.7.1 Measurement of the serum levels of biochemical factors

The morning after the behavioral tests (at 8 a.m.), fasted rats (94th postnatal day) were decapitated under ether for deep anesthesia. The rats were individually placed into a special room to prevent anxiety from the smell of blood. Blood was collected from their jugular vein in tubes without anticoagulant. The samples were then centrifuged at 3000 rpm for 20 min to separate the serum. Corticosterone and leptin levels were measured using standard ELISA kits (Zellbio GmbH Ulm, Germany).

#### 2.7.2 RNA extraction and cDNA synthesis

After homogenizing the hypothalamus immediately after blood sampling, RNA extraction was performed using the RNX-plus reagent (Cinnagen Co., Iran). The extracted RNA was dissolved in 20 μl of DEPC-treated water and the RNA concentration was measured using a Nanodrop™ 2000/2000c spectrophotometer. Subsequently, cDNA synthesis was conducted using a special kit (Parstous, Iran), following the manufacturer's instructions.

#### 2.7.3 Real-time PCR

The mRNA expression of leptin, leptin receptor (LR), glucocorticoid receptor (GR), mineralocorticoid receptor (MR), and GAPDH (internal control) in the hypothalamus, was measured using real-time PCR. Samples were prepared with a combination of SYBR green Master Mix (Parstous, Iran), cDNA, and specific primers for each gene. Real-time PCR reactions were carried out using the SYBR green method in a sequence detection system following the manufacturer's instructions (Qiagen, Germany). The CT value represented the number of PCR cycles needed for the fluorescence signal to surpass the detection threshold. The relative mRNA values for each gene were normalized to the standard gene (GAPDH) value. A final melting curve was performed to check for product specificity and primer dimers. The mRNA levels were calculated using 2−ΔΔCT (Table 2).

**Table 2:** Primers used in the Real-Time PCR.

Primer name	Primer sequence	PCR product size	NCBI accession number
**GAPDH**	F: GTCTTCACCACCACGGAGAAGGC	392	NM_017008
	R: ATGCCAGTGAGCTTCCCGTTCAGC		
**Leptin**	F: CATTTCACACACGCAGTCGG	179	Xm_008762762
	R: GGTTCTCCAGGTCATGAGCTATC		
**LR**	F: CCCAGTTTATGGAAGGAGTTGG	202	NM_012596.2
	R: TTTGGGGTTTGGAACATCGTC		
**GR**	F: ACCCTGCATGTATGACCAATGT	123	NM_003176.3
	R: TTAGGAACTGAGGAGAGAAGCAGTA		
**MR**	F: GATCCAGGTCGTGAAGTGGG	152	XM_039097526.1
	R: AGAGGAGTTGGCTGTTCGTG		

### 2.8 Statistical analyses

The data were checked for normality and then analyzed using Prism 9 software with a one-way ANOVA method and Tukey post hoc test. The results were presented as mean value ± SEM to assess statistical differences between the various groups.

## 3 Results

### 3.1 The influence of different types of stress on anxiety-like behaviors

#### 3.1.1 The elevated plus-maze test

**Fig. 2:**
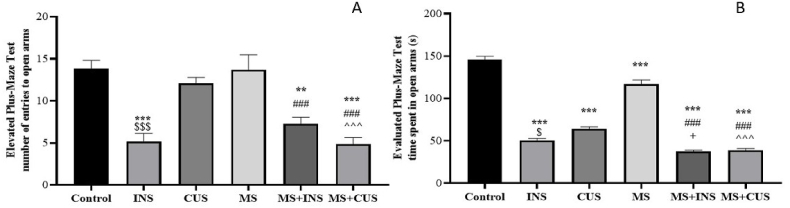
**Elevated Plus-Maze Behavioral Test (EPM).** The changes in the number of entries to open arms (**A**) and time spent in open arms (**B**) following the induction of different types of stress are depicted. Data are presented as mean ± SEM. ∗∗ (P < 0.01) and ∗∗∗ (P < 0.001) indicate significance compared to the control group, ### (P < 0.001) compared to the MS stress group, + (P < 0.05) compared to the INS stress group, ˆˆˆ (P < 0.001) compared to the CUS stress group, ^$^ (P < 0.05) and ^$$$^ (P < 0.001) compared to the INS group. **A**: The number of entries to open arms decreased in the INS, MS + INS, and MS + CUS groups. MS + CUS rats had a lower rate of entries compared to CUS. Additionally, INS rats showed a significant decrease in entries relative to CUS. Both the MS + INS and MS + CUS groups had fewer entries compared to the MS group. **B**: The time spent in open arms decreased in all stress groups. Both MS + INS and MS + CUS groups spent less time compared to the INS and CUS groups, similarly, INS rats showed a decrease in time spent relative to CUS. Both MS + INS and MS + CUS groups had less time spent compared to the MS group.

In this test, the number of entries in open arms showed a decrease in the INS, MS + INS, and MS + CUS groups compared to the control ((P = 0.000) for each group). However, there was no significant difference in the case of the CUS and MS groups. The MS + INS and MS + CUS groups showed a decrease of almost 1.2 times compared to the MS group ((P = 0.025) and (P = 0.000), respectively). Additionally, a remarkable decrease in the number of entries was observed in the comparison between MS + CUS and CUS (P = 0.000). Regarding the time spent in open arms, all stress groups showed a significant decrease compared to the control ((P = 0.000) for each group). The time spent by all stress groups decreased significantly compared to the control ((P = 0.000) for each group). In the comparison between the MS + INS and MS + CUS groups with the MS group, the time spent was less than half ((P = 0.000) for each group). Furthermore, a significant decrease in the time spent was observed in the comparison between MS + INS and INS, as well as between MS + CUS and CUS ((P = 0.035) and (P = 0.000) respectively). In the end, differences between the two adult stress groups were observed for the number of entries (P = 0.000) and time spent (P = 0.011) (see Fig. 2**).**

#### 3.1.2 The open field test

**Fig. 3:**
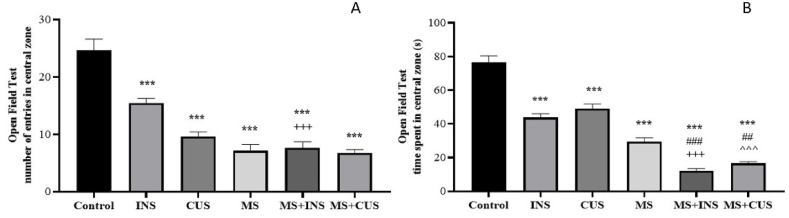
**Open Field Behavioral Test (OFT).** The changes in the number of entries in the central zone (**A**) and time spent in the central zone (**B**) following induction of various stress types. Data are presented as mean ± SEM. ∗∗∗ (P < 0.001) compared to the control group, ## (P < 0.01) and ### (P < 0.001) compared to the MS stress group, +++ (P < 0.001) compared to the INS stress group, ˆˆˆ (P < 0.001) compared to the CUS stress group. **A**: In terms of the number of entries in the central zone, a decrease was observed in all stress groups. MS + INS and MS + CUS rats had a lower rate of entries compared to INS and CUS. **B**: The time spent in the central zone decreased in all stress groups. MS + INS and MS + CUS groups spent less time in the central zone compared to INS and CUS. MS + INS and MS + CUS showed decreased time relative to the MS group.

In this test, the number of entries in the central zone decreased in all stress groups compared to the control group ((P = 0.000) for each group). Additionally, the number of entries in the MS + INS group was lower than in the INS group (P = 0.000). When looking at the time spent in the open arms, all stress groups showed a significant decrease compared to the control group ((P = 0.000) for each group). Furthermore, when comparing the time spent between the MS + INS and MS + CUS groups with the MS group, it was found that the time spent was significantly reduced ((P = 0.000) and (P = 0.003) respectively). Finally, a substantial decrease in the time spent was observed when comparing the MS + INS group with the INS group, as well as the MS + CUS and CUS group ((P = 0.000) for each group) (see Fig. 3**).**

#### 3.1.3 The forced swim test

**Fig. 4:**
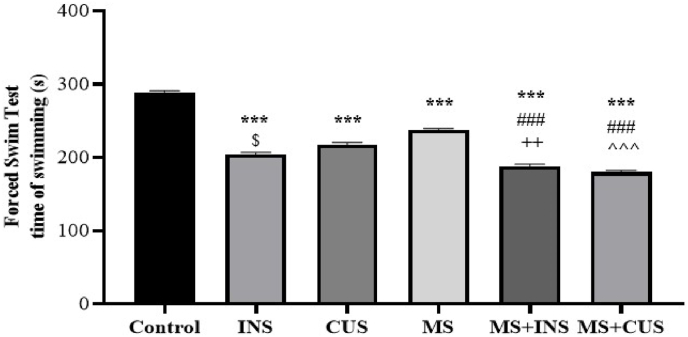
**Forced Swim Behavioral Test.** The changes in swimming time following the induction of different types of stress. Data are presented as mean ± SEM. ∗∗∗ (P < 0.001) compared to the control group, ### (P < 0.001) compared to the MS stress group, ++ (P < 0.01) compared to the INS stress group, ˆˆˆ (P < 0.001) compared to the CUS stress group, ^$^ (P < 0.05) compared to the INS stress group. The swimming time decreased in all stress groups. The MS + INS and MS + CUS groups had a lower swimming time compared to INS and CUS. INS rats also showed a decrease in swimming time relative to CUS. MS + INS and MS + CUS decreased relative to the MS group.

During the forced swim test, we observed a decrease in swimming time among different stress groups compared to the control group ((P = 0.000) for each group). The duration of swimming in the MS + INS and MS + CUS groups was significantly reduced compared to the MS group ((P = 0.000) for each group), and subsequently compared to the INS and CUS groups ((P = 0.004) and (P = 0.000) respectively). Furthermore, the swimming time in the INS group was shorter than in the CUS group (P = 0.015) (see Fig. 4**)**.

### 3.2 Serum levels of biochemical factors (leptin, corticosterone)

**Fig. 5:**
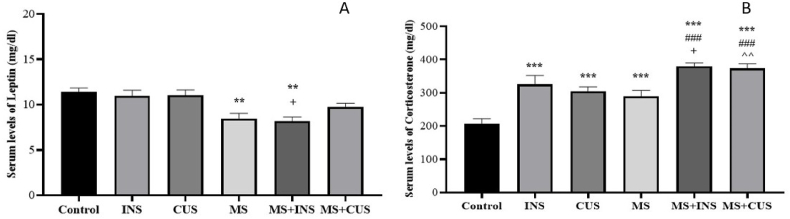
**Serum Levels of Leptin and Corticosteron.** The changes in serum levels of leptin (**A**) and corticosterone (**B**) following the induction of different types of stress. Data are presented as mean ± SEM. ∗∗ (P < 0.01) and ∗∗∗ (P < 0.001) compared to the control group, ### (P < 0.001) compared to the MS stress group, + (P < 0.05) compared to the INS stress group, ˆˆ (P < 0.01) compared to the CUS stress group. **A**: The serum level of leptin decreased in the MS and MS + INS groups. MS + INS rats showed a decrease in leptin levels compared to INS. **B**: The serum level of corticosterone increased in all stress groups. MS + INS and MS + CUS had higher levels of serum corticosterone compared to INS and CUS. MS + INS and MS + CUS increased relative to the MS group.

In Graph A, the serum concentration of leptin decreased in the MS and MS + INS groups compared to the control ((P = 0.009) and (P = 0.003) respectively). Additionally, in the MS + INS group compared to INS alone, there was a decrease in serum leptin concentration (P = 0.013). It is worth noting that the reduction of leptin serum levels in the MS + CUS group compared to CUS did not reach a significant level, but there was a noticeable decrease. In graph B, as anticipated, the serum concentration of corticosterone increased in all stress groups compared to the control ((P = 0.000) for each group). Furthermore, in the MS + INS and MS + CUS groups, there was a remarkable increase in the serum level of corticosterone compared to the MS alone ((P = 0.000) for each group). The comparison between MS + INS and INS, as well as between MS + CUS and CUS groups, showed a significant increase in the serum level of corticosterone ((P = 0.019) and (P = 0.003) respectively) (see Fig. 5**).**

### 3.3 Gene expression

#### 3.3.1 Leptin (L) and leptin receptor (LR)

**Fig. 6:**
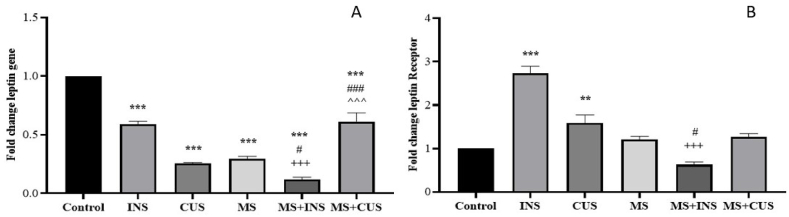
**Leptin and Leptin Receptor Gene Expression in Hypothalamus.** The changes in leptin (**A**) and leptin receptor expression (**B**) in the hypothalamus following the induction of different types of stress. Data are presented as mean ± SEM. ∗∗ (P < 0.01) and ∗∗∗ (P < 0.001) compared to the control group, # (P < 0.05) and ### (P < 0.001) compared to the MS stress group, +++ (P < 0.001) compared to the INS stress group, ˆˆˆ (P < 0.001) compared to the CUS stress group. **A**: The leptin expression decreased in all stress groups. MS + INS rats showed a significant decrease in leptin gene expression compared to INS. Additionally, the expression was higher in MS + CUS compared to CUS. MS + INS had decreased and MS + CUS had increased relative to the MS group. **B**: the leptin receptor expression increased in INS and CUS stress groups. MS + INS showed lower leptin receptor expression compared to INS. MS + INS also decreased relative to the MS group.

In Graph A, hypothalamic leptin expression decreased in all stress groups compared to the control ((P = 0.000) for each group), with the highest decrease in the MS + INS (more than 80%) and MS + CUS (more than 90%) groups. Meanwhile, the level of expression in the INS (up to 40%) and CUS (up to 60%) groups showed a lower decrease compared to the control. Compared to the MS group, MS + INS and MS + CUS showed a decrease in leptin receptor expression ((P = 0.016) and (P = 0.000) respectively). Additionally, there was a remarkable expression decrease in MS + INS and MS + CUS compared to INS and CUS ((P = 0.000) for each group). In Graph B, hypothalamic leptin receptor expression increased in INS (more than 100%) and CUS (up to 50%) groups compared to the control ((P = 0.000) and (P = 0.008) respectively). It is important to note that the expression increase of LR has not reached a significant level in MS compared to the control, but it does show some increase (up to 20%). Also, a strong decrease in LR expression was observed in MS + INS compared to INS and MS groups ((P = 0.000) and (P = 0.011) respectively) (see Fig. 6**).**

#### 3.3.2 Glucocorticoid receptor (GR) and mineralocorticoid receptor (MR)

**Fig. 7:**
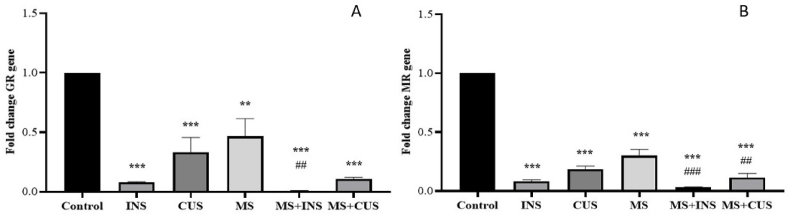
**GR and MR Gene Expression.** The changes in glucocorticoid receptor (**A**) and mineralocorticoid receptor expression (**B**) in the hypothalamus following the induction of different types of stress. Data are presented as mean ± SEM. ∗∗ (P < 0.01) and ∗∗∗ (P < 0.001) compared to the control group, ## (P < 0.01) and ### (P < 0.001) compared to the MS stress group. **A**: The GR expression decreased in all stress groups. MS + INS and MS + CUS rats exhibited lower GR gene expression compared to INS and CUS. MS + INS also showed a decrease relative to the MS group. **B**: The MR expression decreased in all stress groups. MS + INS and MS + CUS groups had lower MR expression compared to INS and CUS. MS + INS and MS + CUS also showed a decrease relative to the MS group.

In Graph A, GR expression was decreased in all stress groups compared to the control ((P = 0.001) for MS and (P = 0.000) for other groups). Particularly, this decrease was observed in the comparison between the MS and MS + INS groups (P = 0.007). Additionally, MS + INS showed a GR expression decrease of more than 10% compared to the INS group and MS + CUS showed a decrease of more than 30% compared to the CUS group. In Graph B, MR expression decreased in all stress groups compared to the control ((P = 0.000) for each group). MR expression was lower in MS + INS and MS + CUS compared to MS ((P = 0.000) and (P = 0.001) respectively). Furthermore, MS + INS showed an expression decrease of more than 10% compared to INS, and MS + CUS showed a decrease of more than 30% compared to CUS (see Fig. 7**).**

## 4 Discussion

The effect of leptin and its receptor in the hypothalamus, one of the main brain centers involved in stress regulation (van Bodegom et al., 2017), has been demonstrated in recent research. Therefore, we measured the expression of leptin and its receptor in the hypothalamus to investigate the mechanisms involved in the effects of prenatal stress and compared these effects in the face of different adulthood stresses.

In the serum corticosterone assay, the results show that stress was well-induced in the mice. Exposure to adult stress in groups with a history of maternal stress caused a significant increase in the activity of the HPA axis. As expected, in rats with elevated serum corticosterone levels, the expression level of GR and MR genes in the hypothalamus decreased. The high level of corticosterone for a long time reduces the mentioned receptors through negative regulation to prevent the destructive effects of corticosterone and HPA axis activity (Gadek-Michalska et al., 2013; Juruena et al., 2020; ter Heegde et al., 2015). According to the results of checking serum leptin levels, unlike acute stress which causes a rapid decrease in serum leptin, chronic adult stress does not affect this factor. However, the reduction of serum leptin in rats with maternal stress is significant. More interestingly, having a history of maternal stress in exposure to INS leads to a greater decrease in serum leptin compared to exposure to CUS. So, in chronic adult stress, serum leptin levels do not change, but cortisol increases. In contrast, having a history of chronic maternal stress decreases leptin and subsequently increases cortisol to a greater extent.

Regarding hypothalamic leptin expression in our results, it is observed that chronic stress causes a significant decrease in gene expression, and among the types of chronic stress, maternal stress shows the greatest decrease. As expected, the history of maternal stress leads to a multifold decrease in leptin expression in the face of adult stress. On the other hand, equivalent to a decrease in leptin expression, we observe an increase in hypothalamic leptin receptor expression in stress groups. This indicates the feedback effect of leptin reduction on the number of receptors and their increase to compensate for the reduction of leptin effects. It has previously been shown that the regulation of leptin receptor expression following leptin expression in different stresses can contribute to proper functioning as a regulatory mechanism (Wauman and Tavernier, 2011). In the meantime, rats with adult stress improved the performance of the leptin system by increasing the expression of the leptin receptor to some extent, but the rats with a history of maternal stress were unable to express more leptin receptors, especially in the face of adult stress, so they experienced more adverse effects as a result of leptin system dysfunction (one of its symptoms is the increase in anxiety-like behaviors, which can be seen in the results of this study).

As shown in animal studies, stress or anxiety increases the transfer of maternal cortisol from the placenta to the fetus (Glover, 2015). Cortisol nuclear receptor (GR) has a non-specific action and can couple with the promoter of more than 500 genes and increase their expression (Weikum et al., 2018). The presence of large amounts of cortisol in the long term creates a phenotype that makes a person susceptible to arteriosclerosis (Dzherieva et al., 2022), cancer (Hong et al., 2021), immune system weakness (Zefferino et al., 2021), heart failure, etc. Recently, it has been shown that in maternal stress, the serotonergic and leptinergic systems of the fetus are affected by cortisol (Groër, 2010; St-Pierre et al., 2016). On the other hand, the HPA axis and leptinergic system interact with each other (Yao et al., 2021).

It has been shown in adrenalectomized ob/ob mice (in which leptin and cortisol are no longer produced naturally), that systemic administration of leptin decreases the amount of CRH (which has increased due to the lack of negative feedback of cortisol) in the PVN nucleus (Roubos et al., 2012). Furthermore, it has been shown that, in obese Zucker rats (less leptin), in response to the chronic increase of glucocorticoid, PVN glucocorticoid receptor content is prominently increased, while in thin Zucker rats (normal leptin) with the same age, not much change is observed (Martins et al., 2022). These observations lead us to conclude that leptin has a direct effect on the control of the HPA axis. On the other hand, leptin probably affects the HPA axis in a dose-dependent manner (Papargyri et al., 2018). The main effects of leptin on the HPA axis are increasing POMC and decreasing CRH (Bouillon-Minois et al., 2021; Chojnowska et al., 2017). It is likely that, in normal doses, leptin has an effect on the PVN nucleus through its receptors and reduces the expression level of CRH and then the activity of the HPA axis (Park and Ahima, 2015). In contrast to this situation, leptin in a low dose affects the ARC nucleus (due to its high receptor density) and increases the synthesis of POMC and, as a result, the activity of the HPA axis (Martelli and Brooks, 2023). On the other side, in acute stress, cortisol directly increases the brain expression of the leptin gene; however, in chronic stress, cortisol decreases its brain expression (Chao et al., 2017). Additionally, glucocorticoids act directly on adipose tissue and change the leptin synthesis, secretion, and central leptin-induced effects (In acute stress, they decrease the leptin serum level) (Ait Eldjoudi et al., 2022).

In addition to the indirect role (through the HPA axis), leptin also plays a direct role in emotional regulation (Endomba et al., 2020). Systemic administration of leptin has been shown to produce anxiolytic effects and removal of the leptin receptor in midbrain dopaminergic neurons results in an anxiety-like phenotype (Fernandes et al., 2021). Inhibition of JAK2/STAT3 signaling in the ventral tegmental area by AG490 attenuates the anxiolytic effect produced by the systemic administration of leptin (Khera et al., 2022; Liu et al., 2016). Also, leptin-deficient mice (ob/ob) show more anxiety-like behaviors in multiple behavioral tests (Watanabe and Sakamoto, 2021).

Lastly, it should be mentioned that, although there is no difference in the biochemical and molecular index between INS and CUS adult stress models, according to the behavior graphs, the CUS model shows better performance. This could indicate a greater effect of social instability stress on anxiety-like behaviors.

## 5 Limitations of the study

This study had limitations, such as a small sample size and only considering male subjects. It would be beneficial to include females in the study and compare the results between the two sexes. Additionally, a larger sample size could help reduce any confounding effects of disordered neural pathways that may be involved in anxiety responses, potentially impacting the creation and quality of anxiety-like behaviors. Ultimately, a larger sample size could lead to more accurate results.

## 6 Conclusion

Based on the results of this research, it can be emphasized that animals experiencing maternal stress are more sensitive and less resilient when faced with adult stress. The changes in serum leptin levels and its impact on hypothalamic function seem to play a significant role in reducing resistance to stress and increasing anxiety-like behaviors in animals exposed to prenatal stress. This is because leptin is involved in modulating the HPA axis and works on various levels to decrease HPA axis activity, ultimately suppressing the stress response. Additionally, it is worth mentioning that when comparing different types of chronic stress, rats subjected to maternal stress are more likely to develop leptinergic system disorders in adulthood, such as anxiety, obesity, and related diseases, compared to other stress groups.

## CRediT authorship contribution statement

**Roya Hosseini:** Writing – original draft, Investigation. **Sara Emadian:** Investigation, Formal analysis. **Manijeh Dogani:** Methodology, Formal analysis. **Touba Ghazanfari:** Funding acquisition. **Nayere Askari:** Writing – review & editing, Supervision, Conceptualization.

## Funding

This study was supported by a grant from the Immunoregulation Research Center of Shahed University.

## Declaration of competing interest

The authors report no conflicts of interest.

## Data Availability

Data will be made available on request.
